# ‘The MRI-scan says it is completely normal’: Reassurance attempts in clinical encounters among patients with chronic musculoskeletal pain

**DOI:** 10.1177/13634593241290185

**Published:** 2024-10-20

**Authors:** Trine CB Andersen, Maja Wilhelmsen, Olaug S Lian

**Affiliations:** UiT The Arctic University of Norway, Norway; University Hospital of North Norway, Norway; UiT The Arctic University of Norway, Norway

**Keywords:** chronic pain, illness perception, patient-clinician communication, reassurance

## Abstract

In clinical guidelines for patients with chronic musculoskeletal pain, reassurance is a key element. The purpose of reassuring patients is to change their views on their illness and, thereby, their actions. However, when symptoms persist without pathological findings, reassurance can be difficult to achieve. Drawing on observations of nineteen naturally occurring hospital consultations with chronic musculoskeletal pain patients, followed by individual interviews with both patients and clinicians, we study how they interact in relation to reassurance. Our main aim is to explore the ways in which clinicians explicitly attempt to provide reassurance, and how patients receive these attempts, before reflecting on facilitating and hindering factors for successful reassurance in relation to the sociocultural context in which their interaction takes place. Through a thematic analysis, four dominating elements of explicit reassurance were identified: (1) education through visualisation, (2) validation through technological findings, (3) validation through physical examination and (4) normalising pain. To gain a deeper understanding of the reassurance process, we then narratively explored dialogical extracts containing these elements. The analysis shows a potential lack of congruence between what patients experience, and the biomedical knowledge clinicians rely on. Despite employing a combination of affective and cognitive modes of reassurance, clinicians tend to build their final conclusions not on patients experiences but on biomedical knowledge, which is knowledge that holds epistemic primacy for themselves. In that sense, their efforts to reassure the patients might also be a way in which they seek to reassure themselves.

## Introduction

In clinical guidelines for patients with chronic musculoskeletal pain (CMP) with unknown pathologies, reassurance is emphasised as an important aspect of care (Oliveira et al., 2018). In a clinical context, the purpose of reassurance is to change patients’ views on their illness by reducing their worries, fears, and doubts and thereby the ways in which they live their lives (Linton et al., 2008). Ultimately, ‘reassurance is achieved if the patient changes his/her behaviour, understanding or thoughts’ (Linton et al., 2008: 5). The reassurance attempts can be related to a wide range of topics, such as aetiology, prognosis, and management, but studies have found a strong link between explanations for symptoms and reassurance (Burton et al., 2015). This means that when symptoms persist without a known pathological cause and positive diagnostic tests, reassurance is difficult to achieve (Burton et al., 2015).

A recent clinical guideline for the treatment of low back pain, a CMP-condition often associated with unknown pathologies, mentions reassurance nineteen times. The guideline states that ‘consistent messaging and *reassurance* from all clinicians involved in a patient’s care are important to dispel myths and support shared decision making. Checking the patient’s understanding of the advice, and whether they *feel reassured*, is essential’. (Australian Commission on Safety and Quality in Health Care, 2022: 11 Authors’ emphasis). Although similar international guidelines for CMP consistently recommend reassurance as an evidence-based treatment option (Corp et al., 2021), there is little clarity on how to deliver such reassurance, or what to do if patients do not feel reassured. The lack of knowledge has long been addressed by researchers in the field (Coia and Morley, 1998; Holt et al., 2015; Linton et al., 2008). These earlier studies have focussed on types of reassurance (affective and cognitive) and their effects (Coia and Morley, 1998; Pincus et al., 2013); patients’ perspective on reassurance (Holt et al., 2015); clinicians’ perspective on reassurance (Giroldi et al., 2014), and reassurance after diagnostic test (Rolfe and Burton, 2013). To our knowledge, no previous studies have observed how reassurance is carried out in consultations, and afterwards collected both patients’ and clinicians’ views on it.

In this paper, we apply this methodology to study how clinicians and CMP-patients interact in relation to reassurance-related elements during clinical consultations. Our study is based on observations of nineteen naturally occurring consultations between CMP-patients and clinicians, followed by post-consultation interviews with both parties. For the clinicians, one of the primary aims of these consultations is to reassure the patients, in the sense of removing their fears or doubts, and motivate them to live their lives as normal as possible. Our main purpose is to explore the ways in which clinicians attempt to provide explicit reassurance, and how patients receive these reassurance attempts, before reflecting on facilitating and hindering factors in relation to the sociocultural structures that influence the reassurance process. During our main analysis, we focus on *explicit* reassurance attempts, meaning those expressed more or less directly. After identifying four dominating explicit reassurance elements, we narratively explore dialogical extracts and identify possible sources of interactional challenges, including the degree of congruences between clinicians’ and patients’ perspectives.

### An interactional perspective on reassurance and its effects

It is estimated that 20%–33% of the world’s population is affected by CMP (Woolf et al., 2012). CMP is defined as ‘ongoing pain felt in the bones, joints and tissues of the body that persists longer than 3 months’ (Booth et al., 2017). It covers multiple conditions with both known pathology (e.g. rheumatoid arthritis) and unknown pathologies (e.g. low back pain) (Woolf et al., 2012). High prevalence, substantial costs for healthcare systems, and work absenteeism positions CMP as a major problem both from a societal and public health perspective (Hartvigsen et al., 2018; Koechlin et al., 2019; Lambeek et al., 2011).

Lack of pathological findings, aetiological explanations, clear-cut diagnosis and predictable prognosis are key issues in the context of CMP. Dealing with these issues, clinicians often focus on reassuring CMP patients through education (Traeger et al., 2015). Previous review studies suggest that cognitive reassurance (education, information) is associated with better outcomes in primary care, and that affective reassurance (empathy, acknowledgement) can improve patient satisfaction (Pincus et al., 2013; Traeger et al., 2015). While the intended emotional (affective) outcome of reassurance, like relief, is immediate, it is only temporary, and research suggests that affective reassurance cannot reduce fears and concerns long term. Cognitive components, on the other hand, is generally found to improve patients’ outcome immediately, and might reassure patients long-term that persisting symptoms are not signs of something dangerous (Pincus et al., 2013). However, some studies have found that the reassurance value of diagnostic tests might be limited (Pienaar et al., 2021; van Ravesteijn et al., 2012), especially when negative (Rolfe and Burton, 2013). Previously, many clinicians assumed that explaining negative tests-results would sufficiently reassure patients, but for patients with unexplained symptoms, this is not enough (Rief and Broadbent, 2007). Burton et al. (2015) argue that in the face of uncertainty and incomplete medical knowledge, it is important to develop explanations which makes sense to both patients and clinicians.

Looking at reassurance in the following analysis, it is placed within a contextual and interactional perspective (Snow, 2008; Strong, 1979). This means we interpret talk and interaction between two parties in relation to its sociocultural context: the clinical consultation in a contemporary western biomedical health system. In this system, patients and clinicians represent different institutionalised positions and the actions of both parties are influenced and constrained by structural and cultural factors on micro, meso and macro levels. Most of all, they are constrained by their different and asymmetrical positions in the social system. The different normative expectations that are attached to their hierarchically structured social roles, and the ‘institutionally specific discursive opportunities’ (Snow, 2008) of the clinical consultation, defines the limits of acceptable speech (Butler, 1997). Traits of the institution itself (a problem-solution format and a technology-dominated biomedical perspective), together with the wider sociocultural surroundings (high expectations of – and faith in – professional knowledge and skills), also impact the ways in which reassurance attempt unfolds. Interpreting our data through a contextual lens means appreciating the impact of the social field in the observed interaction.

## Methods

This study is an interdisciplinary qualitative exploration of interaction in 19 naturally occurring consultations between clinicians and CMP-patients in a specialised hospital rehabilitation clinic in Norway. Data were collected through observations of consultations, followed by post-consultation interviews with both clinicians and patients (12 women and 7 men) immediately after the consultation (Table 1). Observational data, which has been described as ‘the methodological gold standard’ for studying clinical consultations (Timmermans, 2020: 269), enables us to explore how clinical consultations actually are conducted rather than theorised. Post-consultation interviews with all participating parties adds knowledge about how they individually experienced their interaction, which is useful on its own but also in terms of interpreting the observational data.

**Table 1.** Data overview.

Consultation ID	Clinician ID	Duration (min)	Patient ID	Patient age	Patient gender	Pain Area	Interview P (min)	Interview C (min)
C01	C1	92	P1	52	Man	Shoulder	23	29
C02	C2	109	P2	33	Woman	Neck and back	50	48
C03	C3	75	P3	59	Man	Back and shoulder	25	38
C04	C1	64	P4	51	Woman	Back	33	18
C05	C4	108	P5	30	Man	Back	37	43
C06	C1/C5/C2		P6			ME		
C07	C1	69	P7	59	Woman	Hip	22	12
C08	C1	81	P8	29	Woman	Back, neck and head	29	15
C09	C6	130	P9	52	Man	Back, neck and hip	23	41
C10	C1/C3/C7	140	P10	36	Woman	Pelvis and wrists	23	22
C11	C1/C5		P11			ME		
C12	C4		P12			Consultation incomplete		
C13	C3	93	P13	52	Man	Neck	23	23
C14	C3		P14			Above age limit		
C15	C4	30	P15	55	Woman	Numbness in hands	12	13
C16	C2	70	P16	26	Woman	Hypermobility/pain	15	17
C17	C4	63	P17	58	Man	Lower back to foot	53	20
C18	C8	100	P18	30	Woman	Shoulder and lower back	21	37
C19	C8	142	P19	20	Woman	Lower back	13	24
C20	C6 + C7	91	P20	47	Woman	Right hip	29	18
C21	C4	56	P21	49	Man	Lower back and foot	22	19
C22	C2	74	P22	21	Woman	Hypermobility/pain	22	26
C23	C3	54	P23	54	Woman	Lower back to foot	32	16
Average		86		43			27	25

All interviews were carried out the same day, except in C09 where the interview with C3 took place the following day. C6, C11, C12 and C14 are not included in the dataset. The two ME consultations are excluded from the analysis because of the nature and duration of the consultations.

### Participants

Data were collected over 2 months in 2021. The project was presented for the clinical staff, and written consent was collected from all clinicians who wanted to participate (8 of 11 clinicians), including: two doctors, two occupational therapist and four physiotherapists.

Patients were eligible for inclusion in the study if they sought help because of severe long-term pain (minimum 3 months) and were between 18 and 60 years old. If they were not fluent in Norwegian, or had severe learning disabilities, they were excluded. All patients meeting the criteria during the 2-month period were invited, and any patient that responded were included (see Table 1). Because the decision to participate was up to the individual, participants might differ from those who did not respond. The patient group was heterogenic in terms of age, gender, and sociodemographic background. The study strived for an equal gender distribution among patients, but accepted a sample with a small majority of women as this is generally found within this patient group (Bjørneboe et al., 2024).

All study participants were informed that the study focussed on the interaction and communication between clinicians and patients, and informed written consent was obtained prior to the data collection. Patient information, and the procedure of consent, were approved by the local Ethics Committee and the data protection officer (reference no. 807617).

### Observations and interviews

All consultations were observed, audio-recorded and verbatim transcribed. The observer [first author] was sitting in the consultation room as the patient entered and did not participate in the conversation unless asked a direct question. The observer took observational notes filling in the context of the interaction. Immediately after the consultation, the observer would interview the patient and thereafter the clinician. The interviews were individual, semi-structured and in-person. All interviews, which were conducted in the clinic by [first author], aimed at exploring patients’ and clinicians’ respective experiences of the consultations. The semi-structured format allowed flexibility in the interview, so that it could follow the participants narrative by adjusting question sequences and asking open-ended questions.

### Data analysis

Data were analysed in two phases. First phase was based on reflexive thematic analysis (RTA), which is a ‘method for developing, analysing and interpreting patterns’ (Braun and Clarke, 2022: 4). This analytical method was chosen because it is flexible in the way one approaches the dataset. This is important for our study, as it allows our analysis to be informed by previous research, while also working inductively in the coding process. The analysis was done through a systematic process entailing a reading and re-reading of the data. All consultation dialogues were coded in NVivo based on a codebook deriving from coding the first five cases (although some preconceived themes from previous research do appear). The research team includes researchers from sociology, anthropology, and medicine, who during discussion and interpretation of the data, reflected on their own emergent understandings. The initial coding was done by [first author] in collaboration with [last author]. After discussing initial themes, these were reviewed, redefined, and named in a collaborative process that ended with agreement on four main reassurance elements as themes.

In the second phase, consultation transcripts were explored through narrative analysis, at first on a participant-by-participant basis, to respect the uniqueness of each story, and then as a combined dataset. This was done to gain a deeper understanding of the process of reassurance in clinical encounters. Attention was paid to how each story unfolds, and the integrity of the narrative was respected by ‘quoting long extracts and analysing components in light of the whole’ (Lian et al., 2024: 780). By doing so, we seek to preserve the context and meaning of the dialogical data, while capturing the ongoing dynamics of the interactional flow. Because the four reassurance elements often overlap, we present the data not thematically but through three complete consultations. This facilitates a more in-dept analysis allowing us to highlight instances where the four reassurance elements are particularly evident. The three consultations were selected because they together cover all four reassurance elements and captures the main features of the complete dataset. Each of these three cases are headed by quotes from explicit reassurance attempts made by clinicians. In the presentation of quotes, the ‘(. . .)’ indicates an omission of parts of transcripts for anonymisation or brevity and the use of ‘[. . .]’ indicates an addition of information by the authors.

## Results

**Figure 1.**
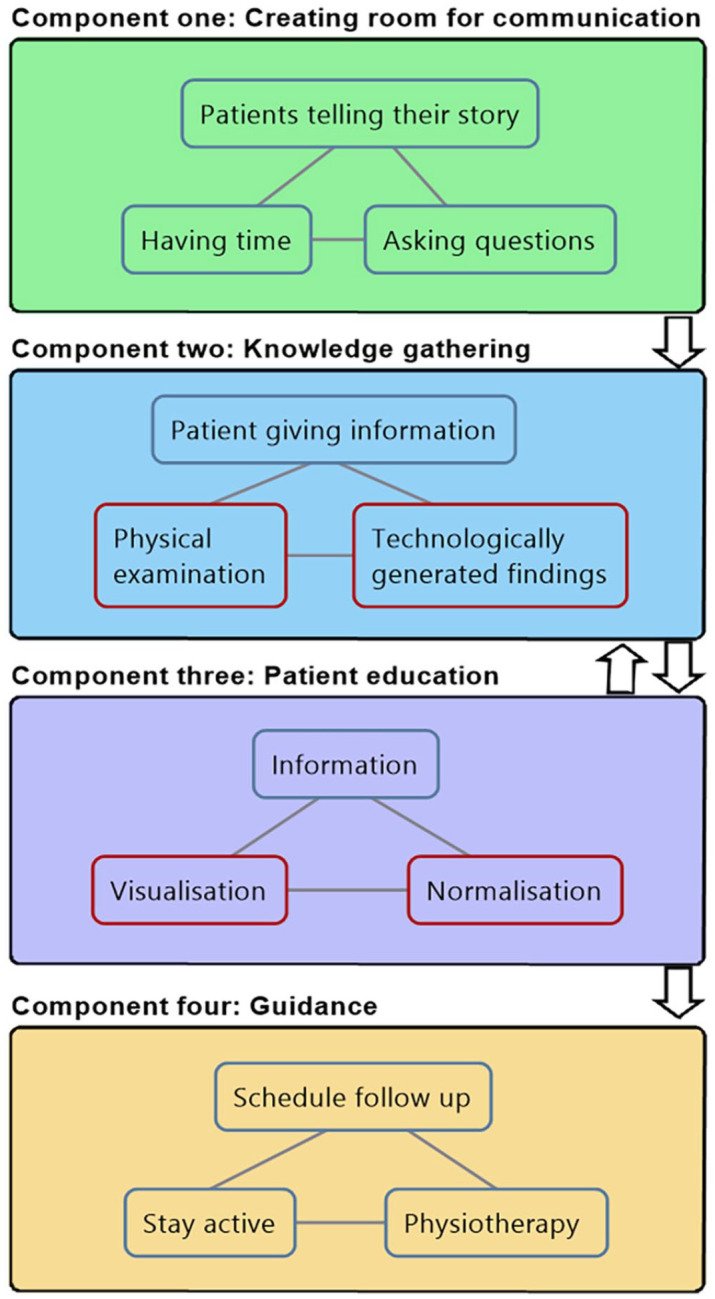
Main components in reassurance attempts. Description: Overview of reassurance components and some key reassurance elements. Red squares indicate the most dominant and explicit reassurance elements. The significance and perception of each element can vary depending on the clinical context and interaction between the patient and clinician.”

Reassurance attempts occurred in a continuous process during the whole consultation, which can be divided into four main components. Initially, clinicians created room for communication (Figure 1, component one) using implicit and indirect reassurance elements such as emphasising ample time, asking questions, and letting patients tell their story. After creating room for communication, the clinicians then started a more targeted gathering of knowledge about patients’ problems through questions and physical examinations (Figure 1, component two), while simultaneously educating them, and normalising their condition (Figure 1, component three). Finally, they provided patients guidance in terms of how to proceed (Figure 1, component four).

The unusual length of our consultations (on average 86 minutes) created special opportunities for affective reassurance attempts (component one), allowing time to build a trusting relationship, which might serve as a foundation for the following more explicit reassurance attempts to be successful. Our focus, however, is on explicit reassurance attempts, as direct statements are more easily identifiable and less ambiguous than indirect and implicit statements. Explicit reassurance attempts were most prominent during knowledge gathering and patient education. Within these reassurance components, we identified four dominating explicit reassurance elements: (1) education through visualisation, (2) validation through technologically generated findings, (3) validation through physical examination and (4) normalising pain. In the following, we discuss these reassurance elements through three separate consultations. Subsequently, we turn to a more detailed analysis drawing on all 19 consultations to reflect on facilitating and hindering factors in relation to the sociocultural structures that influence the reassurance process.

### ‘The MRI-scan says that it is completely normal’

When patients in our dataset arrive at the clinic, many have already seen various clinicians and had multiple technological and clinical test performed. The results from previously performed MRI-scans are commonly referred to by the clinician, without showing the MRI-scan itself, to validate their opinion, and to reassure the patient about the value in what it is ‘saying’, as seen in C08:

C: Uh, that uh I feel confident that it is nothing dangerous because the, uh, the MRI-scan says that it is completely normal.

P: Mhm.

C: Uh, and when I examine you, then I also do not get concerned that it is anything. Although it hurts quite a bit when I touch you.

P: Yes.

C: Yes. But can you live with that?

P: Mm yes ((small laughter)).

C: Now that you know what you know?

P: No, I will be worried.

C: You will?

P: Yes.

The clinician’s reassurance attempt relies on information from the MRI-scan, which ‘says’ that ‘it is completely normal’. By attributing human qualities to a non-human entity (the technology), the clinician avoids being explicit about this being his/her interpretation of the test results. However, the clinician’s statement does not reassure the patient who admits she is still worried. The lack of intended reassuring effect leads the clinician to turn to normalisation:

C: You know that having back pain is very, very common.

P: Mhm.

C: Almost eighty percent of the population will have back pain at some point in their lives.

P: Yes.

C: Yes and very often we don’t know what is causing the pain.

P: Mhm.

C: It is very common that we do not know.

P: Mhm.

C: Ehm, maybe only for fifteen percent can we say that ‘we experience this as the reason for your back problems’.

The clinician here focuses on back pain being a common condition that people experience at some point in their lives, which means that her condition is not unusual or dangerous. The clinician uses the second person plural pronoun ‘we’ as a placeholder for the medical society at large. By doing so, the clinician defers personal responsibility for the lack of answers. In the post-consultation interview, the clinician describes how the patient was afraid that the pain was something dangerous, and the clinician therefore ‘tried to normalise having back pain and [answered] that I had not found any danger signals in the examination and that every single scan, both normal X-ray and MRI, had been normal’. Despite the good intentions, the clinician’s reassurance attempt does not align with the patient’s experiences and beliefs, and she is ‘still worried’.

The patient’s continued affirmations (‘mhm’ and ‘yes’) could suggest acceptance or agreement with the clinician, but in the post-consultation interview the patient states:

‘The [clinician] said quite clearly that it’s nothing serious, there’s nothing like nerve thing or some cancer or something like that but like (.) one should maybe want to get something a bit more specific. (. . .) But if it’s like “yes, but that’s the way it is, and now it’s going to be, maybe, who knows, the rest of my life” then that’s not nice’.

The patient interprets the lack of explanation and the normalisation of pain as being told she just has to live with it, which she resents. Despite feeling this way, she subordinates herself to the clinician through the continued affirmations and performs her role as a patient, as she might think is expected from her. While the clinician’s emphasis on the pain not being dangerous was understood by the patient, she is still worried about what the pain is, and felt that ‘perhaps [the clinician] didn’t realise how much it actually affects’.

The interaction in this consultation illustrates how elements of reassurance take place during consultations. The validation through technologically generated findings occurred in most consultations. This case is indicative of the ways in which reassurance attempts based on technological findings may lead to patients becoming misaligned with the clinician regarding the understanding of their pain-experiences.

### ‘There is nothing to indicate that there is anything dangerous’

After the physical examination, clinicians would often summarise their findings referring to both the technologically generated findings and the physical examination, as in C18:

C: Mm so there is nothing - there is nothing to indicate that there is anything dangerous with this shoulder at all. There is nothing there to indicate that there are any pinched nerves. The picture from September suggests that you had an inflammation in-

P: Yes, yes.

C: -in this one muscle. And it’s also that muscle that’s still a little weak now. When I pushed in, you had the one which just ‘uhh’ come in. So, it is clear there is a slight difference in the strength of your shoulders. Uh, and if you have a slight difference, then how you use it will also be slightly different. (. . .). So, I think that either uhm such patient travel [treatment trip abroad] or that you get a slightly more comprehensive stay so that you can, in a way, take some time for you and your body and try to get things a bit in balance. Because we would like that- ((pause 2 seconds)). I’m out of everything to draw on – right, one thinks that one should- [drawing] If we say, ‘here we’re fine, here we’re not fine at all’.

P: Yes, I feel that I’m down there where I do not feel well at all because I’m in so much pain ((little laughter)).

C: Yes, but I understand that. Of course, then you think ‘okay, they say it’s going to get better’ and you think you should go like this [draws steep line upwards] ‘now I’m going to get better’.

P: Mhm.

C: But the reality-

P: I have realised that I don’t.

The clinician informs the patient that there are no indications of ‘any pinched nerves’, and as in C08, the clinician insist that the patient’s problem is not ‘anything dangerous’. These statements allude to one of the clinicians’ main concerns with the physical examination: to test the patient for what they perceive as ‘dangerous’ conditions such as cancer or pinched nerves. Although patients are likely to perceive such statements as relatively vague (clinicians do not specify what ‘nothing dangerous’ means), the patient responds with brief affirmations (‘mhm’ and ‘yes, yes’), thereby adhering to her subordinate social position by not challenging the clinician’s claims. Furthermore, the clinician uses the MRI-scan to verify and support his/her claims, again attributing it human qualities as it is the picture that ‘suggests’ this interpretation.

To explain why the proposed treatment might be beneficial, the clinician produces a drawing visualising health progression on a scale from ‘not fine at all’ to ‘fine’. In the same manner, the clinician also presents an MRI-scan to visualise what has already been tested, and inform the patient about the lack of findings:

C: (. . .) Anyway, these are the discs that when you get a herniated disc, it’s these that you can see that bulge out. It looks very good, even though this was in September. ((pause 2 seconds)) ((small laughter)) Even though I understand that this is not the answer you wished for.

P: No, I want it to be something wrong with it. ((small laughter)).

C: [cough] Ehm-

P: No, of course I don’t want that but uh. .

C: Yeah, but you want it to be- I can understand because to-

P: Yes, I was happy when I got the arthritis diagnosis. Well, I wasn’t happy, you don’t want to have arthritis, but (.) it took ten kilos off my shoulders because there was actually something wrong. You start wondering if you (.) are a hypochondriac when you have problems for so long.

C: Mhm. (.) Yes but it is difficult- it is difficult to have a disease that you can’t see.

P: Yes.

In this case, the use of the MRI-scan is supposed to reassure the patient that nothing is wrong in her back, by visualising what a herniated disc would look like. However, the clinician recognises that this reassurance attempt does not seem to work, saying: ‘I understand that this is not the answer you wished for’. The clinician thereby pre-empts the patient’s response, which confirms: ‘I want it to be something wrong’. The reassurance attempt does not work as intended, and to deal with the situation the clinician acknowledges the patient’s difficult situation.

In the post-consultation interview, the patient expresses that she was happy with the clinicians drawing about pain as it was: ‘very easy to understand’. However, she feels that the consultation had little impact on her perspective on the future and her pain:

‘(. . .) I was diagnosed with arthritis afterwards and then there was talk of Bekhterev’s and then it wasn’t Bekhterev’s and so now it’s just worse than ever, so I (.) I don’t know if I see the future any brighter right now, but I just have to try to do the things [the clinician] has said and work on recovering’.

**Figure 2.**
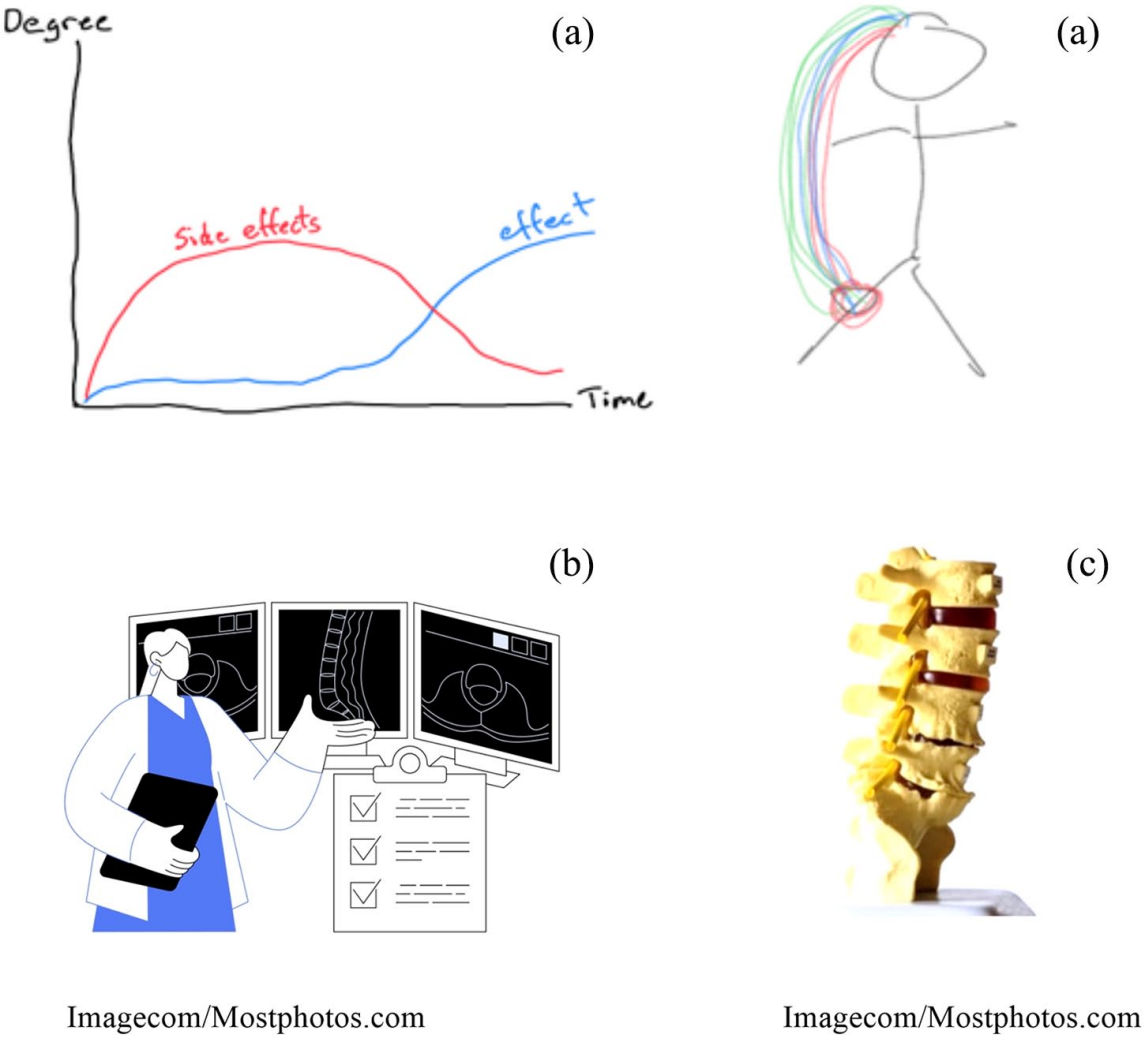
Types of visualisation. Description: The two drawings (a) were made by clinicians to illustrate things such as side effect and effect of medicine (upper left) and pain experience and brains signals (upper right). The MRI-scans (b) were utilised in educating patients about their spine. The herniated disc model (c) was used by clinicians to show what a herniated disc looks like and explain how it works.

The patient’s previous experiences within the health care system, which includes being misled, might have influenced how she received the clinician’s reassurance attempts. While the clinician utilises various reassurance elements to establish rapport and facilitate understanding, for instance by providing clear explanations through visual aids (see Figure 2(a) and (b)), the patient still does not feel reassured about her future.

The presented consultation is indicative of the ways in which clinicians attempted to reassure patients by educating them and visualising what they were explaining. This could for instance be by showing technologically generated findings like MRI-scans (most frequently used), drawing the patient’s body, or using a model of body parts (Figure 2). Post-consultation interviews revealed that most patients were very appreciative of the visualising reassurance element, finding the explanations easier to comprehend. However, it did not necessarily change their understanding of their own problem, as many said their perspective on the future had not changed after the consultation.

### ‘(. . .) it is the findings in the body that are real’

Together with the MRI-scan, results from physical examinations are often used to substantiate clinicians’ account on patients’ problems. In C09, the patient reveals that he would like to be reconsidered for surgery. The clinician, however, does not believe this is the best option for him based on his symptom description and the MRI-scan. To confirm, the clinician performs multiple nerve function tests:

C: At least I think it’s a very good sign that you have such good contact with your feet and-

P: [GP] said that too.

C: Yes.

P: Mhm.

C: So that’s at least a bit of a positive finding then.

P: Yes.

C: Yes. And in the arms too. You have nothing to indicate anything like that, uh - the pictures show that there is tight for the nerve root called C6 that goes out. And that one in a way causes disturbances in the thumb and strength to certain areas of the arm.

P: Mhm.

C: But you don’t have any symptoms of it. So that’s great.

P: Mhm.

C: And so, you can have findings on pictures but, in a way, it is the findings in the body that are real.

P: Mhm.

C: That’s what you kind of do the treatment according to. So had it been surgery that you should have been after, then you would have to have something out in your arm. ((pause 2 seconds)) You do not operate just because of neck pain or (.)-

P: I’ve heard of people who have described some of the [same pain]- someone I play cards with, bridge, he’s had such a [surgery] in [city].

While the clinicians admitted that the physical examination mostly confirmed what they already suspected, they also said that it was performed to reassure the patient, giving them a feeling of being examined. The lack of findings are positive and reassuring to the clinician, as it indicates no surgery is needed. However, saying ‘you do not operate *just because* of neck pain’, the clinician, knowingly or unknowingly, diminishes the patient’s pain experience. After several turns with minimal responses (‘yes’ and ‘mhm’), the patient continues to pursue a biomedical solution by referring to his friend (third party) who had surgery for the same symptoms, thereby indirectly countering the clinician’s professional knowledge and claiming decision-making influence. Such indirect non-confrontational objections, which are commonly observed among patients during clinical interactions, are linked to their subordinate social position in the health system (Lian et al., 2024).

After the physical examination, the clinician summarises the findings using the technologically generated findings as validation:

C: (. . .) You are a little stiff in the back and there are a number of findings [on MRI-scan] that are a bit like wear and tear change that you can imagine causes pain in the back and neck.

P: It’s yeah uhh- like I’ve read it, so it’s this uh so-called fluid that you have in your- spinal fluid or whatever they called it. It was punctured after all –

C: yes right, spinal fluid. I can pull up the pictures if you want.

P: yes. It was dehydrated it said.

C: yes, but I can take the pictures. Because when you look at it, um (.) it’s like (.). Um, do you see the arrows in the back now. If you think this is bone tissue, this is the bones on top of each other. (. . .) Uh, and so what happens over time is that these pillows you have between here, which are a little more elastic than these, because these are completely hard. It’s like bone tissue.

P: Yes.

(. . .)

C: But it is a fairly common finding in everyone.

P: Yes, it was like-

C: So, it is difficult to say that it is painful.

P: No, but there was someone who just said, as if without me being an expert, that you can think of it as a ball joint lubricated with oil, if the oil has dried out.

Using the MRI to visualise the problem, the clinician attempts to educate the patient about what is happening in his back, at the same time attempting to reassure the patient by normalising the findings: ‘but it is a fairly common finding in everyone’. Disregarding this, the patient again indirectly counters the clinician’s professional knowledge by referring to what someone else (third party) told him.

In the post-consultation interviews, when asked what they are most and least satisfied with, the clinician and patient have opposing views. The clinician is most satisfied with the physical examination because ‘I was able to clarify in a way the things I was supposed to do, and tried to inform along the way’. The physical examination validates and supports the clinician’s opinion that the patient does not need surgery. From a biomedical perspective, surgery on the spine, is considered an invasive treatment with high risk and uncertain outcome. Avoiding surgery is therefore presumed preferable and reassuring. Nevertheless, it was the physical examination that the patient was least satisfied with in the consultation, as he has ‘gone through that so many times that it was almost a waste’. To a patient whose whole life is altered because of pain and who has seen other people’s pain alleviated by surgery, the clinician’s conclusion is not reassuring in the sense that it implies no prospects of quick improvements. He concludes: ‘I actually go home with the same problems that I came with, without having become any wiser’. His perspective on the symptoms he is experiencing did not change, and the clinician’s attempt to educate him was not acknowledged.

The presented consultation is indicative of the ways in which clinicians commonly attempted to reassure patients by highlighting null-findings in the physical examination, and how lack of findings was experienced as frustrating and not necessarily reassuring to patients who wanted some kind of medical validation of – and solutions for – their pain.

## Discussion

Four different elements of reassurance attempts dominated our dataset: (1) education through visualisation, (2) validation through technologically generated findings, (3) validation through physical examination and (4) normalising pain. The ways in which these reassurance attempts are expressed and interpreted, and why they represent interactional challenges, has been showcased through three separate cases. In what follows, we discuss the implications of the results in terms of hindering and facilitating factors of reassurance processes.

### Clinicians’ claims about ‘nothing dangerous’

During consultations, clinicians tried to assure patients that their conditions were ‘nothing dangerous’, typically immediately after the physical examination. Expressions indicating absence of danger, might mean that the clinician has established that the patient’s problems are not a symptom of an underlying condition that ought to be treated medically, such as cancer or herniated discs. To validate these claims, the clinicians relied on physical examinations and technologically generated findings, coupled with information about the prevalence of musculoskeletal pain in the world population (functioning to normalise this kind of pain).

These reassurance attempts must be seen in relation to the clinicians’ biomedical perspective. Since the 19th century, Western European medicine has evolved towards what Jewson (2009) names ‘Laboratory Medicine’, which is ‘founded upon the application of the concepts and methods of natural science to the solution of medical problems’. (Jewson, 2009: 625). Within this system, clinical assessments are founded on biological perceptions of health and disease, and a strong reliance on biomedical technology (Jewson, 2009). From a biomedical perspective, the absence of pathological findings on MRI-scans and physical examinations (null findings) indicate that the condition is not dangerous, as well as manageable to live with, given some kind of self-care. Personifying the technologically generated findings, such as the MRI-scans, allowed their conclusions about the absence of ‘danger’ to appear as objective, and independent of personal interpretation. Avoidance of explicit ownership to the interpretation of test results is enhanced by their use of the pronoun ‘we’, to refer to the medical community at large instead of themselves. Both actions – personification and avoiding first-person singular pronouns – share a commonality: the clinicians refrain from taking personal accountability for the conclusion. Deferring personal interpretation and responsibility shield them from potential malpractice allegations and places a patient’s potential dissatisfaction with someone or something else.

Essentially, clinicians were saying what they themselves found reassuring, and what they could confidently rely on. When clinicians focussed on aspects such as null findings and epidemiological data and conclude by saying ‘nothing dangerous’, they might therefore speak to themselves as much as to the patients. Thereby, clinicians attempted to reassure patients about the non-dangerous nature of their pain through knowledge that held epistemic primacy for themselves. This is understandable, given that their professional training, knowledge and judgements safeguard them against clinical errors. However, this also means that clinicians’ efforts to reassure patients, might equally be a way in which they seek to reassure themselves.

### Patient’s response to reassurance attempts

According to The Norwegian Neck and Back Register (NNRR), a vast majority of patients believe that it is pathological structures within the body which causes the pain (NNRR, 2022: 33). These data resonate with the views of patients in our study, who tend to seek biomedical validation of their pain experiences. The patient in C18 explicitly expresses that she wants her pain to be biomedically confirmed (‘I want it to be something wrong with it’), to know that there is ‘actually something wrong’ with her, meaning her experiences are ‘real’. This indicates she believes her own experience is less worth than the medical account of it, and therefore in need of a medical ‘permission to be ill’ (Nettleton, 2006).

Although patients seek biomedical validation, they do not seem to always attribute the same significance to technological and clinical findings as clinicians, rendering these reassurance attempts less effective. Patients thus exhibit a somewhat ambivalent stance towards biomedical knowledge and technological findings: they seek biomedical validation of their symptoms through it, but when no pathological findings appear, they rely more on their embodied experiences than on the null findings. Their initial search for validation must be seen in relation to the high status of the biomedical perspective in the Western European culture, which involves a high level of trust in biomedical knowledge and technology.

In response to clinicians’ reassurance attempts, patients often replied with brief affirmative responses (‘yes’ and ‘mhm’). While this can suggest agreement, our interview data showed that this is not always the case. Patients seem to respond with affirmation, although they disagree with the clinician’s perspective. Additionally, patients hardly ever directly objected to clinicians’ perspectives, but they could object in an indirect non-confrontational way, as seen in C8 where the patient references a third party to indirectly counter the clinician’s professional knowledge and claim decision making influence (Lian et al., 2024). This means that patients act in accordance with their afforded role. In clinical consultation, patients are in a subordinate social position as they, from a biomedical perspective, are perceived less knowledgeable than clinicians, meaning that their experiential knowledge is subordinate to the professional knowledge of clinicians (Pilnick and Dingwall, 2011; Toombs, 1992). Clinicians’ ‘expert status bestows them the authority to override patients’ self-reported experience’ (Buchman et al., 2017: 33) and directly objecting to clinicians would be speaking against those who have the institutionally based authority-position in the clinical consultation. By not voicing their concerns during the consultation, they adhere to their subordinate social position in the clinical setting.

### Hindering and facilitating factors

Although earlier review studies have found that cognitive reassurance, such as educating patients, may have a contradicting impact, it is largely implied that this is the most suitable element of reassurance (Cheung and Soundy, 2021; Holt et al., 2015). This does not include using routine imaging, which guidelines discourage for CMP conditions such as non-specific low back pain (Maher et al., 2017). Despite this, imaging previously performed by other clinicians in the patients’ healthcare journey, plays a significant role for reassurance in our dataset, often serving as validation and as an educational tool.

Alongside various other visualisation forms, MRI-scans were generally acknowledged as a useful educational tool, improving patients’ understanding of the explained phenomena. In contrast, as a validation tool, technologically generated findings seemed to sometimes have an adverse effect. As such, the main difference in perspectives between patient and clinician was found in the interpretation of null findings. Clinicians considered null findings in physical examinations and MRI-scans reassuring. Patients, on the other hand, found null findings discouraging because they represent uncertainty about causes and treatments, and a lack of ‘permission to be ill’ (Nettleton, 2006: 1167). Patients’ initial response to reassurance attempts based on technological and clinical null findings indicate that these arguments have limited impact on patients’ views. Some patients expressed this explicitly, for instance by replying ‘No, I will be worried’ (C08). This is in line with previous research, showing that diagnostic tests hardly make any contribution to the level of reassurance (Rolfe and Burton, 2013; van Ravesteijn et al., 2012).

Component one in the reassurance process (Figure 1), focuses on creating room for communication by giving patients time to describe their symptoms and tell their story. This reassurance component mostly consists of implicit reassurance elements, which is why it is not included in the main analysis. Nonetheless, our patient interviews and findings in other studies (Holt et al., 2015) indicate that this facilitates a trusting relationship between patients and clinicians. Despite that, clinicians in our dataset struggle to utilise this trust during explicit reassurance attempts, in the sense that they sometimes disregard patients’ experiences expressed during component one. In this case, reassurance might not work because the two types of knowledge and perspectives - clinicians’ explanations and patients’ first-hand experiences - are incongruent. Consequently, the clinicians’ efforts might fall short because they do not acknowledge the patients’ personal experiences and knowledge. In the words of Toombs (1992), ‘the decisive gap between lived experience and the scientific account of such experience clash in a direct way with regard to the phenomenon of illness’ (Toombs, 1992: xv).

A shared understanding of the patient’s illness is known to foster patient trust and satisfaction (Lian and Hansen, 2016). We suggest, that while biomedical knowledge might be what the clinicians themselves find most reassuring, it might not be seen as such by patients, given their experiential perspective. The lack of congruence between what the patient experiences and the biomedical knowledge that clinicians rely on, thus complicates the reassurance attempt sometimes leading to adverse effects. Our data shows that when clinicians normalise patients’ conditions and emphasise null findings, validated by technological and clinical test, this can be a hindering factor in terms of accomplishing reassurance. This lack of anticipated effect is understandable, given the different epistemic positions of patients and clinicians (Lian et al., 2024), and their differing perspectives on bodily experiences (Declercq, 2023). One reassurance element (education through visualisation) seems to have the capacity to bridge the gap between patients’ experiences and clinicians’ knowledge and may therefore be a facilitating factor, despite not necessarily changing patients’ perspective on the future.

We acknowledge that our findings do not represent all types of clinical encounters, however the basic interactional elements might occur beyond the study group. We also acknowledge that both patients and clinicians might change their behaviour, knowingly or unknowingly, because they are observed, and their talk recorded. The main strength of this study relates to our multiple and complementary data sources: observations, audio-recordings, and verbatim transcripts of complete consultations, combined with post-consultations interviews with all parties. This gives us a unique insight into how reassurance takes place and is received during naturally occurring consultations.

## Conclusion

While previous studies have investigated types of reassurance and its impact, little has been done to explore how reassurance attempts are carried out and received in consultations. Focussing on four specific explicit elements of reassurance, the findings of this study showcase how reassurance attempts can have various and sometimes adverse effects. We interpret the adverse effects as due to a lack of congruence between what patients experience and the biomedical knowledge that clinicians rely on. The study indicates that clinicians tend to focus on information that is reassuring for themselves and their biomedical background. However, this knowledge might not align with patients’ perspectives. Furthermore, patients’ ambivalent attitude towards biomedical knowledge and technological findings makes it challenging for clinicians to provide reassurance. We suggest that more focus should be on patients’ embodied experiences, knowledge, and perspectives, so that reassurance can be provided on the basis of explanations and assessments which makes sense to both patients and clinicians (Burton et al., 2015).

Clinical guidelines emphasise reassurance as an important aspect of care for patients with CMP. Consultation-based reassurance needs further investigation to understand how reassurance attempts are experienced by patients, and how clinicians should manage situations where reassurance is difficult to accomplish. By gaining a better understanding of how various elements of reassurance work, clinicians will be better equipped to decide how to carry out reassurance attempts. We have addressed this need for knowledge by exploring four explicit reassurance elements, and how they are carried out and received in clinical encounters. This paper also exhibits the importance of looking at the reassurance process through a contextual lens, where the impact of the social field on the clinical interaction is acknowledged.
